# Assessment of COVID-19 Vaccine Acceptance and Reluctance Among Staff Working in Public Healthcare Settings of Saudi Arabia: A Multicenter Study

**DOI:** 10.3389/fpubh.2022.847282

**Published:** 2022-05-30

**Authors:** Muhammad Bilal Maqsood, Md. Ashraful Islam, Ali Al Qarni, Zeb-un- Nisa, Azfar Athar Ishaqui, Naif Khalaf Alharbi, Murtaja Almukhamel, Mohammad Akbar Hossain, Nayyra Fatani, Ahmad Jamal Mahrous, Muhammad Al Arab, Fahad Sami Abdulaziz Alfehaid, Zahida Akbar

**Affiliations:** ^1^King Abdullah International Medical Research Center, Riyadh, Saudi Arabia; ^2^King Saud bin Abdulaziz University for Health Sciences, Riyadh, Saudi Arabia; ^3^Ministry of National Guard Health Affairs, Riyadh, Saudi Arabia; ^4^Department of Pharmacy Practice, College of Clinical Pharmacy, Imam Abdulrahman Bin Faisal University, Dammam, Saudi Arabia; ^5^Department of Medicine, King Abdulaziz Hospital, Ministry of National Guard Health Affairs, Riyadh, Saudi Arabia; ^6^Faculty of Pharmacy, Ziauddin University, Karachi, Pakistan; ^7^Department of Pharmacy, Iqra University, Karachi, Pakistan; ^8^College of Clinical Pharmacy, Imam Abdulrahman Bin Faisal University, Dammam, Saudi Arabia; ^9^Department of Pharmacology and Toxicology, Al Qunfudah Medical College, Umm Al Qura University, Al Qunfudah, Saudi Arabia; ^10^Department of Pharmacy Practice, College of Pharmacy, King Abdulaziz University, Jeddah, Saudi Arabia; ^11^Department of Clinical Pharmacy, College of Pharmacy, Umm Al Qura University, Makkah, Saudi Arabia; ^12^Department of Pharmacy, Prince Sultan Cardiac Center, Ministry of Health, Al Hofuf, Saudi Arabia; ^13^Department of Obstetric and Gynaecology, King Abdulaziz Hospital, National Guard Health Authority, Jeddah, Saudi Arabia

**Keywords:** COVID-19, COVID-19 vaccine, vaccine hesitancy, vaccine acceptance, Saudi Arabia

## Abstract

**Objective:**

The study aimed to evaluate the novel coronavirus disease 2019 (COVID-19) vaccination acceptance and reluctance among staff working in Saudi healthcare facilities.

**Methods:**

A cross-sectional study was conducted during April – May 2021, among healthcare workers in five public hospitals under the National Guards Health Association located in Alahsa, Dammam, Jeddah, Madinah, and Riyadh. The study used a questionnaire in English language, which was distributed through official email communication among healthcare staff currently working at study venues. The data was analyzed using IBM SPSS v23. An ethical approval was obtained.

**Results:**

A total of 1,031 responses were recorded. Most of the staff had both doses of COVID-19 vaccine (89%). The mean score for vaccine acceptance on a scale of 1 (strongly disagree) to 5 (strongly agree) was 3.55 ± 1.6. The mean score for vaccine reluctance on the same scale was 2.71 ± 1.05. Most participants mentioned safety (76.9%) and efficacy (56.3%) as vaccine concerns and believed that COVID-19 vaccine may not be effective because of changes in virus strain (55.5%). The variables of gender and nationality significantly affected vaccine acceptance, while age, gender, nationality, and profession significantly affected vaccine reluctance (*p* < 0.05).

**Conclusion:**

Most healthcare staff were vaccinated, and a high acceptance for COVID-19 vaccination was reported. Several demographic factors affected the vaccine acceptance and reluctance.

## Introduction

The novel coronavirus disease 2019 (COVID-19) pandemic has spread globally and infected millions across the globe, while many have lost their lives due to this infection (1). The world is in the midst of COVID-19 pandemic that is still evolving in terms of its infectiousness and transmissibility. Several new variants of the virus that have high transmission and capability to spread are reported in the scientific literature (2, 3). Besides, the daily reporting of new cases and deaths attributable to COVID-19 is a common occurrence in the news media these days. This has propagated a sense of fear and anxiety among Saudi healthcare workers (4–6).

The emphasis and extensive coverage of COVID-19 in the media and the possibility of early availability of vaccine are unique in this pandemic (1). Besides, the disease has adversely affected the global economy owing to restrictions with regards to social interaction, work, and travel (7). Most of the countries already have strategies to respond to the pandemic crisis, including restrictions on social and large gatherings, travel bans, hand hygiene, and use of face mask. Significant improvement has been observed because of these measures. However, such strategies are not sustainable, and this requires a permanent solution such as medications or vaccines. Efforts are already being made for vaccine development. Therefore, the availability of a COVID-19 vaccine has heightened public excitement (1). It could be said that there is an expectation to return to a normal life post pandemic.

It is important to assess the reaction of healthcare workers toward a novel COVID-19 vaccine as it becomes available. The evaluation of the intent and observed behavior is essential to predict how the recovery from pandemic would take shape. Several studies have been conducted, which strived to report vaccine acceptance among healthcare staff. A study in Indonesia reported that healthcare professionals were more likely to accept a vaccine for COVID-19 (8). In Saudi Arabia, a study reported that >60% of the participants indicated their interest in receiving a COVID-19 vaccine, should it become available (9). However, the study was conducted among the general public and did not analyze the responses from a healthcare subject group specifically.

This study was conducted during the time when the vaccines against the viral infection were approved and prioritized for healthcare staff (10). At the time of this study, the first wave had passed and it was the beginning of the second wave. The healthcare staff were either in the process of receiving a vaccine or had received it. However, receiving vaccination may not be reflective of an individual's acceptance or reluctance as there may be other factors that shape an individual's perception about the vaccine. Such factors may include an individual agreeing to receive a vaccine as a requirement of a purpose such as essential travel during the pandemic. Moreover, some individuals may agree to receive a vaccine as they may believe that it is helpful; however, their confidence may depend on its safety and effectiveness. Therefore, it is important to report the confidence, i.e., acceptance and reluctance in a vaccine for COVID-19, among healthcare staff working in Saudi healthcare settings, as it would not only predict the shape of post-pandemic recovery but also highlight how this confidence would translate into public acceptance in future as healthcare professionals play a pivotal role in providing education and promoting awareness among patients and the general public.

## Methods

### Study Aim

The study strived to document whether the healthcare staff were willing to get vaccinated against COVID-19 disease, and/or report if there was any reluctance to vaccinate. The confidence of the staff was measured through documentation of three traits: the tendency toward registration for a vaccine, the acceptance of a vaccine, and the reluctance toward the same. Therefore, the study aimed to evaluate the confidence of healthcare workers toward COVID-19 vaccination.

### Study Design, Duration, and Venue

This was a cross-sectional study conducted over 2 months, i.e., April – May 2021, at five hospitals under the Ministry of National Guard Health Affairs (MNGHA), across five cities of Saudi Arabia. It included the Imam Abdulrahman Al Faisal Hospital in Dammam, King Abdul Aziz Medical City in Riyadh, Prince Mohammad Bin Abdul Aziz Hospital in Madinah, King Abdul Aziz Hospital in Al-Ahsa, and King Abdul Aziz Medical City in Jeddah. All were tertiary care facilities.

### Study Participants and Eligibility Criteria

The target participants for the study were healthcare staff working at the afore-mentioned venues. The staff who were employed in the above mentioned hospitals and deemed eligible for COVID-19 vaccination as per the Saudi health regulator's COVID-19 vaccination guidelines at the time of study were included. Participants who did not provide consent to participate were not included.

### Sampling Strategy and Sample Size Calculation

The convenience sampling technique was used to collect data from the participants. Participants who had their contact emails available in the list containing organizational emails were contacted. The venue consisted of five public hospitals located in five cities across different regions of the country. It included all the workers of these hospitals. The sample size was calculated using a sample size calculator (11). The margin of error was considered at 3%, while the confidence level was kept at 95%. The required sample size was 1,014. Since the data was collected online, the aim was to gather data more than the required sample size to account for any unforeseen circumstance such as incomplete surveys. An error rate of 10% was included in the final sample. The final sample size was 1,127. The survey analyzed 1,031 complete responses.

### Research Instrument

The research instrument used in this study was a questionnaire. It was developed after review of relevant literature (12–16). Additionally, opinions from practicing healthcare professionals in Saudi healthcare settings were also considered in creating questions. The questionnaire consisted of four sections. The first section was the socio-demographic section that contained questions related to age, gender, marital status, education, nationality, profession, work experience, and workplace. The number of items in this section was 8. The second section contained items related to registration for a vaccine and vaccination status. The number of items in this section was 5. The third section was related to vaccine acceptance and contained 5 items. The last section contained items related to reluctance and concerns and had 8 items.

Mean scores for the acceptance and reluctance toward COVID-19 vaccine were calculated. Items related to vaccine acceptance included belief about importance of vaccine to address the COVID-19, acknowledging the pandemic as a serious health issue in the country, confidence in the accuracy of a vaccine, willingness to get vaccinated upon availability of a COVID-19 vaccine, and willingness to vaccinate family members upon availability of a COVID-19 vaccine. Items related to vaccine reluctance included reluctance to vaccinate, concerns about the possible adverse effects, and concerns about the rushed pace of vaccine development overlooking potential adverse effects. All items were designed as Likert scale from 1 to 5, where 1 meant strongly disagree while 5 meant strongly agree. A mean score was calculated from these items. Some items were dichotomous, i.e., contained a Yes/No response, and were not included in scoring.

The questionnaire was available in English language as it was the primary means of communication among the employees at the study venues. The questionnaire was also piloted on 15 participants before the actual study. Healthcare professionals, academicians, and students participated in the pilot study. The instrument was piloted on 7 pharmacists, 3 medical practitioners, 3 academicians, and 2 pharmacy students. All participants, except students, had at least 3 years of work experience. No difficulty in understanding of the questions was observed. The pilot data was not included in the actual study.

### Data Collection

Data for the study was collected from the staff using the questionnaire. The survey was encoded by the data management section of the institute using Lime Survey platform, in a weblink, and was distributed *via* email through the corporate communication office of MNGHA. Several email reminders were sent later using the same staff list to increase the response rate to achieve the desired sample size. The data collected was anonymous, and the respondents could not be identified from their responses.

### Data Analysis and Management

Data analysis was done through IBM SPSS program version 23. The descriptive statistics such as mean, median, and standard deviation (SD) were used for reporting continuous data, while frequency (%) and sample counts (N) were used to report categorical data. The variables of “vaccine acceptance” and “vaccine reluctance” were the dependent variables. Simple and multiple linear regression analyses were employed to report the significance predictors of vaccine confidence. The level of significance was 5%.

The data was without any personal identifiers, and the data file was password protected. It was sent through official communication and stored in a password-protected computer. Any hardcopies created during analysis were securely disposed.

### Ethics Approval and Consent

The study was approved by the Institutional Review Board at the King Abdullah International Medical Research Center (KAIMRC), Saudi Arabia, on 10th April, 2021. The study number was NRA21A/015/03 and the memo reference number was IRBC/0804/21. The approval was applicable to all healthcare facilities. The questionnaire was filled through an email link sent through official communication. The survey was accessible to participants after they reviewed the study consent section and agreed to participate voluntarily.

## Results

A total of 1,031 responses were analyzed. Most of the staff were aged between 41–50 years (*N* = 409, 39.7%) and had an experience between 10 and 15 years (*N* = 244, 23.7%). Most were females (*N* = 750, 72.7%), non-Saudi (*N* = 747, 72.5%), married (*N* = 668, 64.8%), and had a bachelor's degree (*N* = 751, 72.8%). More than half were nurses (*N* = 681, 66.1%) (Table 1).

**Table 1:** Demographic characteristics of study participants (*N* = 1,031).

**Characteristics**	**Frequency (N)**	**Percent (%)**
Age		
20–30	166	16.1
31–40	399	38.7
41–50	409	39.7
>50	57	5.5
Gender		
Male	281	27.3
Female	750	72.7
Marital Status		
Single	312	30.3
Married	668	64.8
Divorced	38	3.7
Widowed	13	1.3
Education level		
Bachelor	751	72.8
Masters	110	10.7
Doctorate	170	16.5
Nationality		
Saudi	284	27.5
Non-Saudi	747	72.5
Occupation		
Doctor	293	28.4
Nurse	681	66.1
Pharmacists	4	0.4
Allied Health	40	3.9
Support Staff	13	1.3
Work experience (years)		
1–5	167	16.2
6–10	223	21.6
11–15	244	23.7
16–20	171	16.6
>20	226	21.9
Healthcare facility		
Imam Abdulrahman Al Faisal Hospital	32	3.1
King Abdul Aziz Medical City	628	60.9
Prince Mohammad Bin Abdul Aziz Hospital	60	5.8
King Abdul Aziz Hospital	137	13.3
King Abdul Aziz Medical City	174	16.9

The majority (*N* = 935, 90.7%) registered themselves on the web application for vaccination, while more than half (*N* = 697, 67.6%) strongly agreed that they were willing to register immediately upon announcement. Slightly more than a third of participants (*N* = 337, 32.7%) registered themselves on the web application between 1 and 3 months. Majority had taken an influenza vaccine (*N* = 811, 78.7%) and both doses of COVID-19 vaccine at the time of data collection (*N* = 918, 89%) (Table 2).

**Table 2:** Response distribution for vaccine registration and vaccination items (*N* = 1,031).

**Items and response**	**Frequency**	**Percent**
I have registered myself for COVID-19 vaccination at “Sehaty or MNG-HA” Application^*^		
No	96	9.3
Yes	935	90.7
I was willing to register for vaccination immediately when it was announced		
Strongly disagree	47	4.6
Somewhat disagree	48	4.7
Neither agree nor disagree	79	7.7
Somewhat agree	160	15.5
Strongly agree	697	67.6
Timing to register for vaccination when it was announced		
Less than 1 month	330	32
1–3 M	337	32.7
4–6 M	175	17
7–9 M	45	4.3
More than 9 M	144	14
I have taken Influenza vaccine in last 12 months		
No	220	21.3
Yes	811	78.7
I have taken first dose of COVID-19 vaccine		
No	35	3.4
Yes	996	96.6
I have taken both doses of vaccine^**^		
No	113	11
Yes	918	89

* *At the time of survey*.

** *No represents one dose taken and/or no dose taken*.

For the participant's view of vaccine acceptance, the mean score was 3.55 (3.45–3.65 for 95% confidence interval [CI], 1.60 SD). The Cronbach's alpha value of the items was 0.979 that highlighted an acceptable reliability. The mean score for several items related to the COVID-19 vaccine acceptance are mentioned in Table 3.

**Table 3:** Novel coronavirus disease 2019 (COVID-19) vaccine acceptance among staff (*N* = 1,031).

**Items**	**Mean (95% CI of Mean)**	**SD**
I believe that vaccine is important to combat the COVID-19 pandemic	3.60 (3.50, 3.71)	1.70
I think that COVID-19 pandemic is a serious health condition in Saudi Arabia	3.60 (3.50, 3.71)	1.71
I am confident about accuracy of COVID-19 vaccine.	3.42 (3.33, 3.51)	1.52
I am willing to get vaccinated immediately upon availability of COVID-19 vaccine	3.57 (3.46, 3.67)	1.70
I will vaccinate my children/spouse/family members if vaccine is available immediately	3.55 (3.44, 3.65)	1.71

For the participant's view of vaccine reluctance, the average mean score of the three items related to the COVID-19 vaccine reluctance was 2.71 (2.65–2.78 for 95% CI, 1.05 SD). The Cronbach's alpha value of the items was 0.715 that highlighted an acceptable reliability. The mean score for several items related to the COVID-19 vaccine acceptance are mentioned in Table 4.

**Table 4:** COVID-19 vaccine reluctance among staff (*N* = 1,031).

**Items**	**Mean (95% CI of Mean)**	**SD**
I am reluctant to get COVID-19 vaccine	1.80 (1.73, 1.88)	1.28
I am worried about possible side effects of a vaccine for myself	3.21 (3.13, 3.30)	1.35
I am worried that the rushed pace of testing the new COVID-19 vaccine may have failed to detect potential side effects or dangers	3.12 (3.04, 3.21)	1.34

Further, most participants mentioned safety (*N* = 700, 76.9%) and efficacy (*N* = 580, 56.3%) as vaccine concerns. Most participants sought additional information regarding COVID-19 vaccine, such as compatibility with health conditions (*N* = 529, 51.3%), and safety and reliability of vaccine (*N* = 660, 64%). Slightly more than half of the participants believed that COVID-19 vaccine may not be effective because of changes in virus strain (*N* = 572, 55.5%) (Table 5).

**Table 5:** COVID-19 vaccine concerns among staff (*N* = 1,031).

**Concerns**	**Responses**	
	**Yes** **(N & %)**	**No** **(N & %)**
**I have following specific concerns(s) about the vaccine**		
Safety (e.g., Side effects)	700 (76.9)	331 (32.1)
Efficacy	580 (56.3)	451 (43.7)
Newness, including not wanting to be the first to get the vaccine	352 (34.1)	679 (65.9)
Vaccine contents	383 (37.1)	648 (62.9)
No concerns	200 (19.4)	831 (80.6)
**I need additional information about vaccine for my satisfaction**		
Compatibility with personal health conditions (e.g., allergies, comorbid condition	529 (51.3)	502 (48.7)
Recommendation from doctor or officials	280 (27.2)	751 (72.8)
Timing regarding state of pandemic, personal immunity	406 (39.4)	625 (60.6)
Safety and reliability of vaccine	660 (64)	371 (36)
I do not need additional information	233 (22.6)	798 (77.4)
**I believe that COVID-19 vaccine is not effective because**		
Change in virus strain	572 (55.5)	459 (44.5)
Hastiness in vaccine development	140 (13.6)	891 (86.4)
Rush in Vaccine testing process	277 (26.9)	754 (73.1)
Less information available about safety of vaccine	313 (30.4)	718 (69.6)
All of above	268 (26)	763 (74)

The model for COVID-19 vaccine acceptance revealed that gender and nationality were significant predictors after adjusting other variables. Males reported higher likelihood mean score for acceptance. The acceptance score increased by 0.78 (*p* < 0.05) when other factors are adjusted. Besides, on comparison based on nationality, i.e., Saudi vs. non-Saudi, the likelihood score for acceptance decreased by 0.154 for Saudi citizen (*p* < 0.05), provided other variables are considered. The variables of bachelor of education, and all professions except allied health were found significant in simple regression analysis only. All other variables such as level of education, marital status, profession, and work experience were non-significant when adjusted for demographic characteristics of participants (Table 6).

**Table 6:** Model for COVID-19 vaccine acceptance among staff (*N* = 1,031).

**Characteristics**	**Simple Regression**		**Multiple Regression**		
	**Coefficient (β)**	***p*-value**	**Coefficient (β)**	***p*-value**	**VIF**
Age (in year)					
≤ 40 vs. >40	−0.013	0.679	—	—	—
Gender					
Male vs. Female	0.122	<0.001	0.078	0.041	1.55
Nationality					
Saudi vs. Non-Saudi	−0.175	<0.001	−0.154	<0.001	1.11
Marital status					
Single vs. Married	0.049	0.114	—	—	—
Bachelor education					
Yes vs. No	0.141	<0.001	0.076	0.111	2.41
Masters' education					
Yes vs. No	−0.045	0.147	—	—	—
Doctorate education					
Yes vs. No	−0.131	<0.001	−0.024	0.609	2.39
Physician (Profession)					
Yes vs. No	−0.127	<0.001	0.026	0.573	2.34
Nurse (Profession)					
Yes vs. No	0.132	<0.001	^*^	^*^	^*^
Allied Health (Profession)					
Yes vs. No	−0.023	0.453	—	—	—
Work Experience (in years)					
≤ 15 vs. >15	0.010	0.757	—	—	—

* *Removed from model due to Multicollinearity problem, Multiple regression model applied. Model fitness tested by: ANOVA (F = 10.078, p = < 0.001); R^2^ = 0.047 and adjusted R^2^ = 0.042*.

Simple regression revealed that except for the master level of education, all variables including participants' age, nationality, marital status, bachelor and doctorate levels of education, professions (physician, nurse, and allied health), and work experience were significantly associated with reluctance toward COVID-19 vaccine. The multiple model for COVID-19 vaccine reluctance revealed that for a change in age group from ≤ 40 years to > 40 years, the reluctance score increased by 0.094 (*p* < 0.05), provided other variables are constant. Besides, considering gender, compared to females, the reluctance score increased by 0.079 (*p* < 0.05) for males, when other factors are considered. Further, while considering the nationality of participants, the reluctance score increased by 0.070 (*p* < 0.05) for Saudi participants compared to non-Saudis, when adjusted for participant's demographics. Moreover, for profession, the reluctance score decreased to 0.108 (*p* < 0.05) for physicians when compared to non-physicians, when all other demographic factors are considered. On the contrary, the reluctance score increased by 0.072 (*p* < 0.05) for allied health profession compared to others while adjusting for participant's demographics (Table 7).

**Table 7:** Model for COVID-19 vaccine reluctance among healthcare staff (*N* = 1,031).

**Characteristics**	**Simple Regression**		**Multiple Regression**		
	**Coefficient (β)**	***p*-value**	**Coefficient (β)**	***p*-value**	**VIF**
Age (in year)					
≤ 40 vs. >40	0.130	<0.001	0.094	0.045	2.73
Gender					
Male vs. Female	0.139	<0.001	0.079	0.041	1.60
Nationality					
Saudi vs. Non-Saudi	0.062	0.048	0.070	0.044	1.28
Marital status					
Single vs. Married	0.086	0.006	0.027	0.421	1.17
Bachelor level of Education					
Yes vs. No	0.085	0.006	−0.047	0.338	2.56
Masters level of Education					
Yes vs. No	−0.013	0.665	—	—	—
Doctorate level of Education					
Yes vs. No	−0.091	0.003	−0.021	0.664	2.40
Physician (occupation)					
Yes vs. No	−0.150	<0.001	−0.108	0.031	2.67
Nurse (occupation)					
Yes vs. No	0.096	0.002	^*^	^*^	^*^
Allied Health (occupation)					
Yes vs. No	0.098	0.002	0.072	0.030	1.18
Work Experience (in year)					
≤ 15 vs. >15	0.094	0.003	−0.014	0.763	2.18

* *Removed from model due to Multicollinearity problem. Multiple regression model applied. Model fitness tested by: ANOVA (F = 5.935, p = < 0.001); R^2^ = 0.050 and adjusted R^2^ = 0.041*.

## Discussion

It could be argued that vaccines are perhaps among the strongest measures that could help mitigate the risk of the COVID-19 infection and its resultant impact on the daily lives. Vaccination against the viral infection could help reduce its spread, thereby reducing the likelihood of reversing the preventive measures that impact daily life. This large-scale multicenter study was conducted to document the confidence of staff working at healthcare facilities of Saudi Arabia, regarding vaccination against COVID-19 infection.

It was observed that most of the staff were quite positive toward vaccination, as more than 90% mentioned that they registered themselves for vaccination through the web application as soon as it became available. At the time of data collection, almost 90% of the respondents had taken their second dose. In this context, a study among healthcare workers in the US reported that out of every 20 participants surveyed, 3 were found to be hesitant (17). On the other hand, another study in the same population in Germany reported a vaccine acceptance of 91% (18).

Secondly, the respondents showed good acceptance of COVID-19 vaccine, as the average mean score for the items regarding the same was 3.55 out of 5. In this context, a study among healthcare workers in the neighboring country of the UAE reported that vaccine acceptance was high (>89%) (19). Similar finding was reported from the same population in Kuwait (20). The staff shared their opinion that vaccine was important in addressing the pandemic, and acknowledged it as a serious issue in the country. Several studies conducted among the general population of Saudi Arabia reported an increased readiness to vaccinate, and most participants held positive perceptions about the vaccines. However, a sizeable portion of the population also showed their reluctance with concerns regarding safety (21). Another study conducted among a small sample of healthcare workers in Saudi Arabia highlighted that 50% were willing to receive a vaccine, out of which roughly 49% seemed willing to receive it immediately upon availability (22). Another study reported an acceptance of roughly 65% (23). However, the timeline of data collection for both studies was up to December 2020. Our study has been relatively recent and highlights that this acceptance greatly increased and literally doubled in the following year. Such an occurrence shows the increase in confidence of healthcare staff toward vaccination.

The Organization for Economic Cooperation and Development (OECD) mentions that public trust in vaccines against COVID-19 is as important as the effectiveness of the vaccines, and the actions of the governments to increase this trust could be a determinant for their success (24). According to published sources, Saudi health authorities approved the use of vaccine for preventing COVID-19 as early as December 2020 and prioritized geriatrics and healthcare workers to receive the vaccine (25). Later, two more vaccines were approved for use (23). Moreover, the health authority launched the web applicaton to register for receiving a vaccine. The receipients were able to book a date as early as 24 h (26). Such measures were pivotal in increasing the uptake of vaccines by the residents. Hence, these might be the reasons as to why there was an increase in acceptance compared to previous studies. However, this change also points to the fact that such opinions toward vaccination have been largely fluid and may not be consistent. Therefore, it is imperative that such measures are continued to ensure that acceptance remains consistent or improves further.

Further, it was reported that the average mean score for reluctance toward a COVID-19 vaccine was 2.71 out of 5. Although it was low, and given the fact that 90% of the participants received a vaccine, it still cannot be ignored. A high mean score >3 was observed for the statement regarding worry about adverse effects of vaccine. This apprehension was also reported by participants in previous studies (21, 23). Moreover, another statement with a high mean score for reluctance >3 was about the failure to detect dangerous adverse effects due to the rushed pace of vaccine development. This occurrence was also witnessed as health regulators found rare adverse effects such as blood disorders and myocarditis as a consequence of receiving COVID-19 vaccines (27, 28). To this end, a study in Qatar reported that a small proportion of healthcare workers, roughly 13%, had vaccine hesitency (29).

An important finding was that more than half of the participants were of the view that the vaccine may not remain effective owing to the mutations that occur in a circulating virus. The healthcare workers in Qatar also had doubts over vaccine's protection (29). According to the World Health Organization (WHO), the currently available vaccines may not become completely ineffective in the face of emerging variants and would continue to offer reasonable protection against these new variants. However, it is imperative that measures are taken to reduce the spread so as to reduce the likelihood of the virus to mutate into a new variant (30).

There is a massive drive for vaccination in MNGHA hospitals. The organization had a dedicated vaccination center in each hospital for staff at the time of writing. Therefore, vaccine related information is readily availabile and accessible. The availability of vaccine is ensured within the hospital. This study had a limitation. It was not possible to estimate the response rate and at the same time, considering the online nature of study, the response is usually low. Several email reminders were sent to overcome the issue of a low response rate. We estimate that our response rate was lower than 70%.

## Conclusion

The findings of this study reveal that most participants were vaccinated and expressed confidence in COVID-19 vaccination. Some of the apprehensions such as adverse effects and effectiveness of vaccines on variants of COVID-19 virus were genuine and were true in retrospection. Several demographic factors affected the vaccine acceptance and reluctance.

## Data Availability Statement

The datasets presented in this article would be available from corresponding author upon suitable request. Requests to access the datasets should be directed to AL, azfar.hd@hotmail.com.

## Author Contributions

MM: conceptualization, interpretation and writing—original draft, revision, and editing. MI: conceptualization, methodology, analysis, validation and writing—review original draft, revision, and editing. AA: conceptualization, interpretation and writing—original draft, revision, and editing, proposal review, ethics review process, critical review of manuscript, and feedback. ZN: writing, critical review of manuscript, revision, and feedback. NA: methodology, writing, critical review of manuscript, and feedback. MA: writing, results, critical review of manuscript, and feedback. MH and AM: conceptualization, methodology and writing—review, and editing. NF: conceptualization, interpretation, critical feedback, and editing. MAA and FA: proposal review, ethics review process, critical review of manuscript, and feedback. ZA: conceptualization, interpretation, methodology, results, and writing—original draft and editing. All authors agreed on the final version for submission.

## Conflict of Interest

The authors declare that the research was conducted in the absence of any commercial or financial relationships that could be construed as a potential conflict of interest.

## Publisher's Note

All claims expressed in this article are solely those of the authors and do not necessarily represent those of their affiliated organizations, or those of the publisher, the editors and the reviewers. Any product that may be evaluated in this article, or claim that may be made by its manufacturer, is not guaranteed or endorsed by the publisher.
